# Partner-assisted cognitive behavioural therapy for insomnia versus cognitive behavioural therapy for insomnia: a randomised controlled trial

**DOI:** 10.1186/s13063-019-3334-3

**Published:** 2019-05-08

**Authors:** Alix Mellor, Kellie Hamill, Melissa M. Jenkins, Donald H. Baucom, Peter J. Norton, Sean P. A. Drummond

**Affiliations:** 1 Monash Institute of Cognitive and Clinical Neurosciences, School of Psychological Sciences, Monash University, 18 Innovation Walk, Clayton, VIC 3800 Australia; 2 The Center for Stress and Anxiety Management, San Diego, USA; 3 Department of Psychology and Neuroscience, University of North Carolina, Chapel Hill, USA

**Keywords:** Insomnia, Couples, Bed partner, CBTI, Sleep, Partner-assisted interventions

## Abstract

**Background:**

Insomnia is a prevalent sleep disorder associated with significant economic and personal burdens. Cognitive behavioural therapy for insomnia (CBTI) is considered the gold standard intervention for insomnia and its efficacy has been well demonstrated. However, the core treatment strategies of CBTI require significant behavioural change, which many individuals find challenging. As a result, although CBTI is efficacious, its effectiveness is reduced by modest levels of adherence in typical clinical settings. This is problematic as adherence is essential to attain desired treatment outcomes. Sleep is often a dyadic process, with approximately 60% of Australian adults sharing a bed. Hence, the present study aims to determine whether incorporating bed partners into treatment for insomnia increases treatment adherence and completion. The impact of adherence on symptoms of insomnia will also be examined.

**Methods:**

This study is a mixed-effects randomised effectiveness trial of partner-assisted CBTI (PA-CBTI). It is an “effectiveness” (as opposed to “efficacy”) trial, due to the focus on “real world” clinic-based clients and adherence/attrition as outcomes. Participants will include 120 clients with insomnia who are randomly assigned, in equal numbers, to PA-CBTI, traditional individual CBTI (i-CBTI), or partner-assisted sleep management therapy (PA-SMT; which serves as the control group). All interventions consist of seven weekly 1-h sessions. Treatment outcome is evaluated using clinician-rated treatment adherence, and diary-based adherence to stimulus control and sleep restriction. Clients and partners complete major assessments at pre- and post-treatment, and at 6-month follow-up. Secondary outcome variables include actigraphy, self-report measures related to sleep, comorbid psychopathology, and relationship functioning.

**Discussion:**

This is the first randomised clinical trial to examine the impact of incorporating the bed partner in the treatment of insomnia. Results will provide new information about the role partners play in clients’ insomnia presentation and treatment response, and better define the role of adherence in CBTI. This trial has the potential to optimise treatment outcomes for insomnia by improving adherence and reducing attrition. Results could have far-reaching impacts. Improvements in insomnia have been linked to improvements in mental and physical health and, given the high financial costs of insomnia, this study could have a positive economic impact.

**Trial registration:**

ACTRN, ACTRN12616000586415. Registered on 5 May 2016.

**Electronic supplementary material:**

The online version of this article (10.1186/s13063-019-3334-3) contains supplementary material, which is available to authorized users.

## Background

### The impact of insomnia

Insomnia is a highly prevalent and impairing sleep disorder, affecting 10–15% of adults worldwide [1–5]. In Australia, 34.5% of the adult population report regular difficulty falling asleep or staying asleep, hallmark symptoms of insomnia, and many develop a diagnosable insomnia disorder [6]. Insomnia is associated with substantial personal and economic costs. Individuals with insomnia report lower quality of life [7], are at greater risk of accidents [8–11], and have high rates of psychiatric comorbidities [1]. For example, in Australia from 2009 to 2010, half of the clients seen for depression in General Practitioners’ offices also presented with insomnia [12]. In addition to depression, individuals with insomnia are more likely to develop a range of mental health issues, including suicidality, substance abuse, and post-traumatic stress disorder (PTSD), as well as physical health problems such as hypertension, diabetes, and cardiac events [6]. Insomnia carries substantial economic burden ($10.9 billion in Australia in 2010) due to high healthcare costs, reduced work productivity, and increased accidents both at work and on the road [6].

### Behavioural interventions for insomnia

There is a strong evidence base for the non-pharmacological treatment of insomnia [13, 14] with behavioural intervention [15]. Cognitive behavioural therapy for insomnia (CBTI) has been identified as the gold standard intervention for treating insomnia. CBTI is a multi-component intervention based on Speilman’s behavioural model of insomnia [16]. CBTI is efficacious in the short-term; individuals maintain therapeutic gains at long-term follow up, and research indicates its long-term superiority to pharmacotherapy in several randomised controlled trials [17, 18]. Treatment effects are seen in primary and comorbid insomnia [19]. Furthermore, CBTI produces greater subjective and objective sleep improvements when compared with no treatment, progressive relaxation therapy, and pharmacologic and non-pharmacologic placebo interventions [20]. CBTI also has a number of practical advantages: it is relatively brief (i.e. seven to eight sessions on average), can be delivered effectively in multiple formats (e.g. individual or group [20]), and is generally preferred by clients over medications [18].

### The importance of adherence and attrition

Dismantling studies have shown that the two components carrying the most variance in treatment outcomes in CBTI are sleep restriction therapy (SRT) and stimulus control (SC) [21]. SRT requires the individual with insomnia to stay up much later and/or get out of bed much earlier than they typically do; this builds sleep pressure, in essence by sleep depriving clients. SC requires getting out of bed when the individual is unable sleep, something many individuals find very difficult in the middle of the night. Both strategies are challenging and require significant effort to maintain adherence. Therefore, while CBTI is efficacious, as with any behavioural intervention, it only produces the desired outcome when the individual adheres to the treatment recommendations and completes the treatment. Although the challenges of sleep restriction and stimulus control are typically overcome in tightly controlled randomised controlled trials (RCTs) due to the added support provided by the study and the typically homogenous sample, they pose a challenge for mainstream clinicians without those added resources and enrolment restrictions.

The two places where the potential difficulties associated with the behavioural changes demanded by CBTI would be expected to diminish outcomes in more real-world settings are: 1) adherence to the treatment protocol; and 2) attrition (i.e. treatment drop-out).

A recent review shows that adherence to CBTI in the clinical settings ranges from 32–52% [21]. The lowest rates were in clinical effectiveness studies and studies with comorbid populations [21]. Not surprisingly, even modest improvements in CBTI adherence can improve outcomes [22]. Findings from a study of 696 clients with insomnia found that those who completed treatment with high adherence improved significantly more than completers with low adherence [22]. Additionally, adherence is the strongest predictor of long-term (e.g. 1 year) outcomes [22]. Attrition also reduces the benefits of treatment, especially in clinical settings where dropout rates are reported to be as high as 39% [23, 24]. A study on the national dissemination of CBTI in the US Veteran Affairs Healthcare System reported that 33% of clients dropped out [24]. Together, these studies suggest a sizable proportion of clients are likely not to be fully benefiting from CBTI. Efforts to broadly implement CBTI would benefit from ways to improve treatment adherence and completion rates. There currently are no evidence-based recommendations for augmenting either treatment adherence or completion in CBTI.

### Partner-assisted interventions: increasing adherence and completion

One attractive option for increasing adherence and completion in CBTI is via the involvement of bed partners due to 60% of Australian adults sharing a bed. A growing body of literature supports the effectiveness of couple-based interventions for treating individual psychiatric disorders including mood, obsessive compulsive disorder, PTSD, eating disorders, and substance-related disorders [25–28]. Within the sleep field, qualitative studies have suggested including the bed partner in continuous positive airway pressure treatment for sleep apnoea [29, 30]. In “partner-assisted” interventions, partners play a supportive role by acting as surrogate therapists, helping clients make behavioural changes outside of the therapy setting [31]. Treatment focuses on improving the client’s symptoms without directly targeting the couple’s relationship [32]. Partners in this role are taught to help provide an environment favourable to behaviour change and to reinforce positive client changes [32]. This is important, because well-intentioned partners can negatively impact clients’ psychiatric conditions and treatment gains [32]. For example, caring partners can inadvertently accommodate clients’ maladaptive behaviours in an effort to demonstrate support, avoid conflict, or decrease distress for clients or themselves. Such accommodation can often reinforce maladaptive behaviours and work against treatment goals [32]. Therefore, the need to educate partners may well be relevant in insomnia.

To our knowledge, the only partner-assisted intervention for insomnia to be tested was performed by the investigator team during the treatment development phase prior to the current RCT. Thus, this study aims to determine whether enlisting partner support in adhering to difficult behaviour change, and teaching partners to reduce ineffective, albeit well intentioned, accommodating behaviours, could serve to optimise important environmental and behavioural conditions in the treatment of insomnia.

### Couples and sleep

The literature on couples and sleep is relatively sparse, especially in the context of insomnia. However, recent reviews and the few observational studies in this domain highlight several important points. Importantly, sleep is often a social experience since the majority of adults have bed partners [33]. Consistent with this idea, couples prefer to sleep in the same bed and report worse sleep when they sleep alone, despite objective evidence for negative consequences on sleep from co-sleeping [34]. Moreover, couples influence each other’s sleep. For example, Lee et al. found sleep duration covaried within couples on a daily basis, and sleep quality covaried within couples when averaged across several days [35]. Sleep is also related to relationship satisfaction. For example, Troxel et al. found sleep disturbance, including insomnia, and relationship satisfaction were associated [33]. Similarly, those with sleep-onset insomnia were more likely to report relationship problems as a result of their own or their partner’s sleep issues [36]. Another study found higher marital satisfaction was linked to higher sleep-wake concordance in couples [37], and a large study (*n* = 405 couples) found a spouse’s sleep problems predicted higher levels of marital unhappiness [38]. Finally, couple-level variables, including bed partner behaviours, can perpetuate insomnia [34]. For example, bed partners can encourage a client to sleep in or take a nap after a bad night of sleep, or to read, watch TV, etc. in bed until they become sleepy, or they can increase a client’s attentional preoccupation with sleep by asking about their sleep in the morning. While these behaviours are generally well-intended, they are all antithetical to treatment. Therefore, evidence suggests that partners affect each other’s sleep and relationship satisfaction, and thus incorporating a partner into CBTI has the potential to significantly impact outcomes. Surprisingly, though, no published studies report development or testing of a CBTI intervention incorporating the partner.

## Research aims and hypotheses

Finding ways to maximise treatment effectiveness in insomnia is of paramount importance. The present study’s broad aim is to increase adherence to and completion of CBTI. We will endeavour to do this using partner-assisted CBTI (PA-CBTI), a novel strategy which incorporates the bed partner in the intervention. We will systematically assess the value added by including the partner in the intervention. PA-CBTI will be compared with two control treatments (individual CBTI (i-CBTI) and partner-assisted sleep management therapy (PA-SMT)). The present study’s hypotheses are based on the premise that: (a) including the partner will increase adherence to treatment recommendations; and (b) the active ingredients of CBTI are required for good clinical outcomes. Thus, we expect the partner-assisted interventions to show better adherence, the CBTI interventions to show better outcomes, and only PA-CBTI to show both.

### Aim 1

To examine the impact of adding the partner to treatment on adherence and completion.

#### Hypothesis 1A

Participants in the partner-assisted treatment will show better treatment adherence to SRT and SC, measured by daily sleep diaries and greater overall adherence on clinician-rated adherence forms, relative to those receiving individual CBTI.

#### Hypothesis 1B

Participants in the partner-assisted treatment will evidence higher treatment completion rates than i-CBTI.

### Aim 2 (secondary outcomes)

To examine the impact of adherence on insomnia symptoms in the short- and long-term (6 months post-treatment completion). This will test if adherence is directly related to outcomes and if the combined effects of the partner and CBTI are superior to either alone.

#### Hypothesis 2A

In PA-CBTI and i-CBTI, but not PA-SMT, adherence will be positively associated with diary-based sleep efficiency at post-treatment assessment.

#### Hypothesis 2B

Due to increased adherence, sleep outcomes will be better at 6-month follow-up in PA-CBTI relative to i-CBTI.

## Methods

**Fig. 1:**
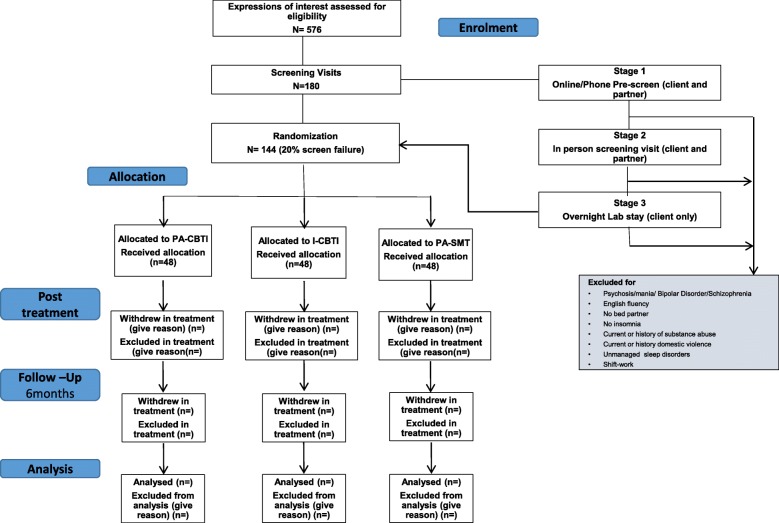
Standard Protocol Items: Recommendations for Interventional Trials (SPIRIT) diagram for enrolment, allocation, follow-up, and analysis. Participant numbers noted are those anticipated, not those currently enrolled/completed. CBTI cognitive behavioural therapy for insomnia, I individual, PA partner-assisted, SMT sleep management therapy

This study is a mixed-effects randomised effectiveness trial of partner-assisted CBTI (PA-CBTI). It is an “effectiveness” (as opposed to “efficacy”) trial, due to the focus on “real world” clinic-based clients and adherence/attrition as outcomes. Participants are randomly assigned, in equal numbers, to PA-CBTI (the active treatment group), or to one of the two control conditions: traditional individual CBTI (i-CBTI) or partner-assisted sleep management therapy (PA-SMT). A flow chart of enrolment, allocation of treatment, and follow-up is presented in Fig. 1.

### Participants

Study participants (*n* = 120 completers) are clients and their partners recruited from the Monash Healthy Sleep Clinic (Melbourne, Australia) and the community via advertisement, e.g. radio, newspaper, flyer drops, and social media. Our estimated recruitment numbers shown in Fig. 1 are based on a 4:1 referral-to-screening ratio, and our initial screening visit goal assumes a 20% screen failure rate. Our randomisation numbers assume 20% attrition. These rates are similar to those we have experienced in other similar insomnia trials.

### Inclusion and exclusion criteria

Participant eligibility criteria are outlined in Table 1. We considered carefully whether to exclude participants taking sleep medications but decided against this based on the recommendations of Morin et al. [42]. We are, however, tracking medications throughout the study.

**Table 1:** Eligibility criteria for participants

Inclusion	Exclusion
a) Adults (> 18 years of age) b) Diagnosis of insomnia disorder, per DSM-5 c) A stable bed partner willing to participate in the study (i.e. someone with whom the client has lived and shared a bed ≥ 5 nights/week for ≥ 1 month d) Fluent in English	a) Unmanaged psychosis or manic episodes in the past 2 months in either partner b) Untreated sleep disorders other than insomnia (managed sleep disorders such as obstructive sleep apnoea treated with continuous positive airway pressure will be allowed) c) Current substance-use disorder (SUD), or SUD within the last 90 days d) Current or history of domestic violence e) Shift work f) Travel across time zones in the past month prior to commencing treatment g) Pregnancy after the first trimester h) An infant < 1 year of age

There are no exclusion criteria related specifically to the partner, other than exclusions a, c, d, and h

We considered carefully whether to exclude participants taking sleep medications, but decided against this based on the recommendations of Morin et al. [42]. We will, however, track medications throughout the study

### Screening and eligibility

When participants first express interest in the study, they complete a brief pre-screening questionnaire either online or via telephone. This questionnaire is administered to both clients and partners and asks questions pertaining to sleep, mental health, time travel, shift work, and relationship status.

At the initial visit, and following written informed consent, a 2- to 3-h study eligibility screening and pre-treatment assessment is conducted. This assessment involves: (a) Duke Structured Interview for Sleep Disorders (DUKE; [39]) to assess insomnia and other potential sleep disorders (client only); (b) Structured Clinical Interview for DSM-5 (SCID-5; [40]) to document any other psychiatric disorders (client only); (c) medical and sleep history; and (d) a series of self-report measures as shown in Table 2. Participants also undergo an overnight polysomnography to rule out excluded sleep disorders.

### Study intervention

Following pre-treatment assessments, couples are randomised to one of three interventions: PA-CBTI, i-CBTI, or PA-SMT. All three interventions consist of seven weekly, 1-h sessions. Participants can discontinue the treatment or the study at any stage. If one of the members of the couples wishes to withdraw, they must withdraw as a couple. Post-treatment and 6-month follow-up data will be collected for those who discontinue the treatment unless they withdraw from the study.

#### Cognitive behavioural therapy for insomnia (CBTI) and partner-assisted CBTI (PA-CBTI)

We employ a CBTI protocol based on those recommended by the leading experts in the field [40, 41]. This protocol covers the four-factor model of insomnia, sleep restriction, stimulus control, sleep hygiene, stress and relaxation, cognitions and sleep, and relapse prevention. PA-CBTI follows the same session outline as CBTI. The “partner-assisted” aspect involves: (a) teaching the partner all of the same concepts and psychoeducation as provided to the client; (b) providing handouts of session material tailored specifically for the partner; (b) actively engaging the partner in all treatment-related decision making and problem-solving; and (d) actively engaging the partner in determining how they can assist the client to implement treatment recommendations at home and assigning those behaviours as the partner’s homework. For example, we engage the partner along with the client in reviewing sleep diaries of the client, identifying patterns, deciding on changes to make, and determining whether/how to titrate time-in-bed for the coming week. The partner also actively assists the client to pick bed/wake times, activities and locations related to stimulus control principles, and strategies for staying awake and/or avoiding naps, etc. In essence, anything a client does in traditional CBTI is done with input from the partner in PA-CBTI. The therapist emphasises empowerment of the partner to provide support and structure for the client at home to maximise adherence to the intervention.

#### Partner-assisted sleep management therapy (PA-SMT)

Sleep management therapy includes all aspects of CBTI, except sleep restriction therapy and stimulus control: the 3P model of insomnia, sleep hygiene, stress and relaxation, cognitions and sleep, and relapse prevention. We use the 3P model in PA-SMT because it does not include conditioned arousal as the fourth factor, as this factor is intimately tied to stimulus control. While the primary goal of this study is to compare PA-CBTI with i-CBTI regarding adherence and attrition, inclusion of a non-active control arm facilitates interpretation of results. Specifically, PA-SMT will help control for the interaction of non-specific therapy factors and the involvement of the partner. We expect this condition to show high adherence without conferring benefits to sleep. This is also a valuable control because sleep hygiene (the core of SMT) is the only treatment many individuals with insomnia are offered given that CBTI is typically is only available in specialty clinics. If results indicate that adding the partner increases adherence (as expected) and this, in turn, increases sleep outcomes, PA-SMT would be a much more cost effective way of treating sleep problems than i-CBTI or PA-CBTI. To be clear, we do not expect that outcome, but it would be remiss not to examine for it. In PA-SMT, a different topic is covered in each session, including education about sleep-wake cycle, sleep hygiene, exercise, diet, alcohol, and caffeine. Sleep restriction and stimulus control principles are strictly avoided. Prior studies have shown that SMT is a credible and well-accepted intervention, though it does not improve sleep [43, 43].

### Fidelity

Manuals are used in all treatment sessions with the psychologist to ensure treatment fidelity, and specific training is provided to all clinicians for all manuals. To ensure fidelity to the delivery of treatment, all sessions are audio recorded. Treatment fidelity is emphasised in several ways: (a) therapists receive intensive training on all interventions; (b) therapists receive weekly group supervision, including the supervisor listening to selections of that week’s audio recordings; and (c) audio recordings of every session from the first two cases of each treatment and 10% of all subsequent sessions are evaluated for treatment fidelity (based on standardised check lists of session content).

### Outcome measures

#### Primary outcome measures

Assessments include: (a) major assessments conducted pre- and post-treatment and at 6-month follow-up for both the client and partner; and (b) weekly and daily assessments for the client as shown in Table 2.

**Table 2:**
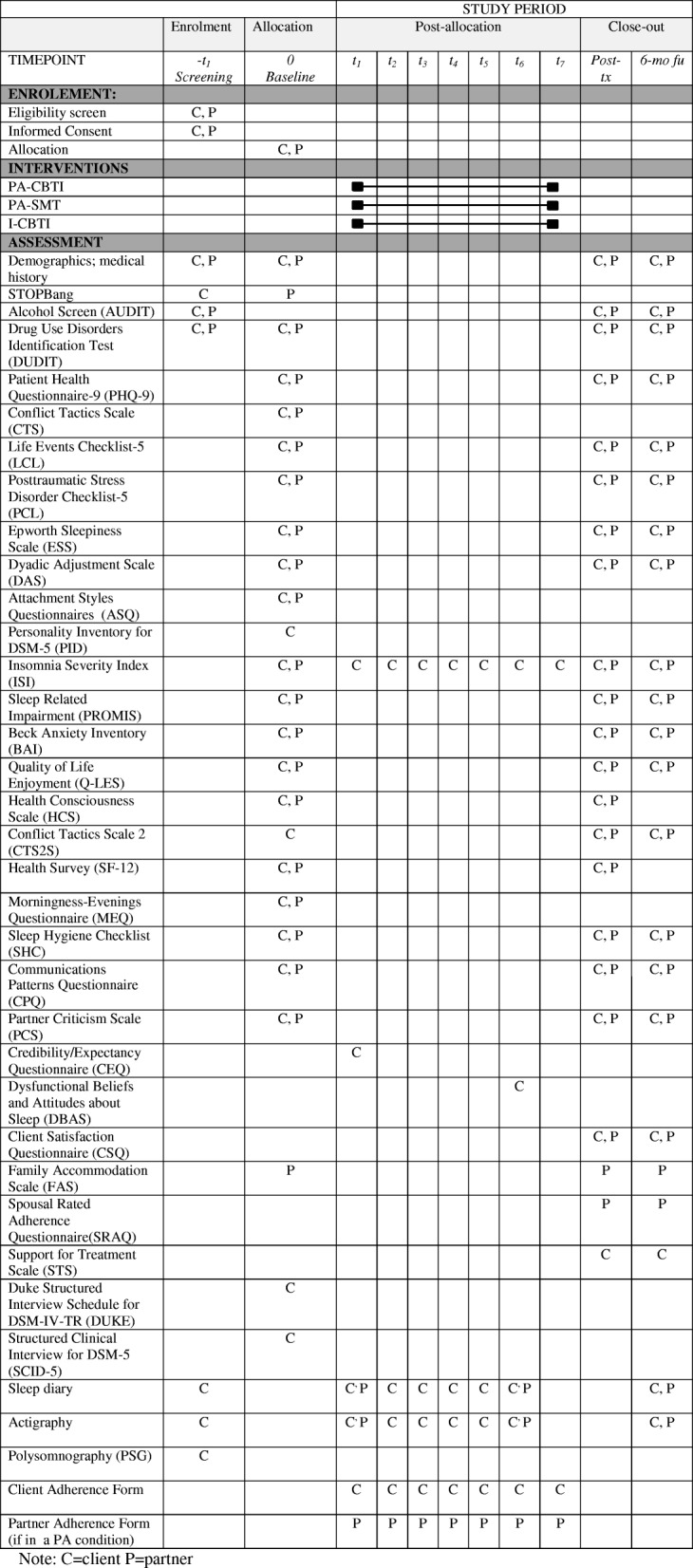
Schedule of enrolment, intervention, and assessments for Project REST. *C* client, *CBTI* cognitive behavioural therapy for insomnia, *fu* follow-up, *I* individual, *P* partner, *PA* partner-assisted, *SMT* sleep management therapy, *tx* treatment

Primary outcome variables include treatment adherence to sleep restriction and stimulus control, as assessed by sleep diaries, clinician-rated adherence forms, and treatment completion rates.

##### Sleep diaries

Sleep diaries are widely used in studies of insomnia [44]. Each morning, participants are asked to record sleep habits such as bedtime, wake time, time in bed, and number and duration of awakenings and naps. Weekly averages are calculated. Sleep diaries include questions to track treatment adherence. Adherence to sleep restriction is calculated as average nightly deviation, in minutes, between the prescribed bed/wake times and reported times, plus any nap time [41, 45]. Note that, consistent with sleep restriction, clients are not considered “non-adherent” if they go to bed later or wake up and get out of bed earlier than scheduled times as they are taught this will simply increase sleep debt and aid treatment. Stimulus control adherence involves two questions: the first asking how many wake minutes after lights-out were spent out of bed (we then calculate that as a proportion of total wake minutes), and the second asking how many minutes outside the sleep window were spent in bed doing any activity other than sleep. Sleep management therapy adherence is measured by documenting the extent to which the treatment recommendation(s) to be practised each week is followed. Additionally, adherence to sleep restriction and stimulus control will still be calculated, despite these not being treatment recommendations. Hence, this group serves as a control for how much these behaviours change without a specific focus on them. While having several measures of adherence poses analytical challenges, the advantage is it allows us to assess exactly where, if anywhere, adherence fails. Adherence measures are obtained in sessions 3–7 (changes to sleep commence at session 2). We considered also using actigraphy to assess adherence (i.e. objective measure of bed time/wake time in sleep restriction). However, we decided against this, since actigraphy cannot measure stimulus control (i.e. cannot determine whether motion is in or out of bed) or assess sleep hygiene.

##### Client adherence form (CAF)

The CAF was created for the US Veterans Affairs CBTI training program using an expert consensus process. Using a scale of 1 to 6 (no adherence to complete adherence) or not applicable (for strategies not yet taught), therapists rate client adherence on six specific CBTI activities [22]. A modified version has been developed for this trial, including for PA-SMT. Clinicians complete this form after each treatment session every week.

#### Secondary outcome measures

##### Sleep measures

The Insomnia Severity Index (ISI) consists of seven items assessing severity of insomnia in the past week [46, 47].

Using the sleep diary, diary-calculated sleep efficiency, time spent awake after sleep onset, sleep onset latency, and early morning awakenings are secondary outcome variables. These variables are calculated both as weekly averages and as night-to-night variability measures.

Actigraphy, an objective measure, is not part of the diagnostic criteria for insomnia. However, these data are commonly reported in insomnia studies and will provide data for secondary analyses. Participants wear the Actiwatch Spectrum Pro (Philips Respironics) either throughout treatment as well as for 1 week prior to the 6-month follow-up (clients) or for 1 week at each assessment time point (partners). Actiwatch data are collected in 30-s epochs in its standard sensitivity wake threshold (score wake at more than 40 activity counts per epoch) and scored using the Cole-Kripke algorithm. Weekly averages are calculated.

##### Relationship measures

The Dyadic Adjustment Scale (DAS; [48]) is a widely used 32-item inventory with sound psychometric properties of relationship adjustment in couples.

The Communication Patterns Questionnaire (CPQ; [49]) is a widely used 23-item self-report measure assessing couple communication before, during, and following discussion of relationship problems.

##### Comorbid psychopathology

The Patient Health Questionnaire-9 (PHQ-9 [50]) is a 9-item questionnaire assessing severity and frequency of depression symptoms.

The Posttraumatic Stress Disorder Checklist-5 (PCL-5 [51]) consists of 17 items assessing trauma symptoms based on DSM-5 criteria.

The Beck Anxiety Inventory (BAI [52]) has 21 items assessing general anxiety symptom severity.

The Quality of Life Enjoyment and Satisfaction Questionnaire Short Form (Q-LES-Q-SF; [53]) is a 16-item self-report questionnaire assessing quality of life on multiple domains.

Each of these measures are given during major assessments.

##### Treatment expectations and satisfaction

Since client and/or therapist expectations could impact outcomes, both will complete expectation measures after the first session using the Treatment Credibility/Expectancy Questionnaire. Data will be examined as a possible mediator of outcomes.

The Client Satisfaction Questionnaire (CSQ; [54]) is an 8-item questionnaire that provides a measure of client satisfaction with services received.

#### Allocation to treatment conditions

Participants are randomised to one of three treatment conditions. Randomisation occurs within clinicians such that each clinician sees an equal number of clients in each treatment condition. Randomisation occurs separately for male and female clients to help ensure equal male:female ratios for each treatment condition.

#### Blinding

Blinding of participants and clinicians is not possible due to the different structure of the treatment interventions. However, clients are blind to the study hypotheses and outcome variables. Staff who score polysomnography and actigraphy are blind to the treatment condition and hypotheses. There are no other “observer-rated” assessments at post-treatment or 6-month follow-up.

#### Attrition analysis

Analysis of non-completers will be assessed based on those who were scheduled for an initial treatment session. We document the reasons for attrition throughout, and will assess the difference between completers and non-completers, as well as the differential attrition rates between treatment conditions. This forms part of the secondary hypothesis given completion is one of the main outcome variables.

### Data management and processing

#### Data management

Data are submitted to a secure university server. Participants are allocated a unique identification code that is used for all study records to maintain confidentiality and protect their identity. All treatment sessions are audio recorded for fidelity purposes. Audio recordings are labelled by the clinician and saved on the secure server at the end of each session.

#### Power considerations

We have powered the study for Hypothesis 1A. Hypotheses 1A tests the outcome (i.e. adherence) related to what makes PA-CBTI unique from traditional CBTI. Given the relatively small sample size of our pilot PA-CBTI data (unpublished), we have benchmarked our power analysis to find a medium effect (Cohen’s f = 0.25) in a 3 × 5 (group × session) multivariate analysis of variance (MANOVA) (five sessions, as there are no adherence data in weeks 1 or 2). With alpha = 0.05 and a two-tailed test, a sample size of 120 (40 per group) provides a power of 0.81 to detect a significant effect. Follow-up comparisons also have adequate power [55].

#### Statistical tests of hypotheses

##### Aim 1

Hypothesis 1A predicts that individuals under partner-assisted conditions will show greater adherence and lower attrition than those under the individual treatment condition. We will test this with 3 × 5 (group × session) MANOVA. The MANOVA will first test the entire set of adherence variables by constructing a latent variable of adherence and using this as the outcome variable. If significant, we will then examine the univariate results for each specific adherence measure. Significant main effects of group (the hypothesised effect) will be followed by comparing PA-CBTI with each of the other groups. Hypothesis 1B will use the Chi-squared test to test the proportion of participants who complete each treatment, with similar follow-ups.

##### Aim 2

Hypothesis 2A anticipates that greater adherence will relate to better sleep outcomes in PA-CBTI and i-CBTI, but not PA-SMT. Multiple regressions will test the hypothesis, with explanatory variables of group (dummy coded), adherence (in the last week of treatment), and group × adherence interaction. The outcome variable will be diary-derived sleep efficiency in the last week of treatment. The interaction term is the effect of interest. Follow-ups will examine the adherence effect within each group. Hypothesis 2B will be tested with Sobel mediation. We hypothesise that PA-CBTI will show (via sleep diaries) greater mean daily sleep efficiency at post-treatment and 6-month follow-up compared with i-CBTI. This effect will be mediated by adherence at the end of treatment (adherence cannot be measured at follow-up since we will not be prescribing sleep schedules).

##### Exploratory analyses

Any large study such as this produces a plethora of data useful for analyses not included in the primary and secondary aims. This study is no exception. Examples of such possible analyses from this protocol include: a) the impact of the sleep interventions on sleep in the partner, particularly that subset of partners who show clinically relevant sleep symptoms at baseline; b) dyadic analyses of sleep examining how each individual in the couple affects the other’s sleep and whether this changes with treatment; c) impact of the interventions on relationship satisfaction, mental health symptoms, and quality of life; and d) impact of sleep interventions on objective measures of sleep as assessed via actigraphy.

#### Publications

All results will be published. Trial results will be communicated to participants via email. Publications will be written by members of the research team. Publications are planned corresponding to the main hypotheses and aims of the study: (a) to examine the impact of including the partner in treatment on variables of adherence and completion; and (b) to examine the impact of adherence on insomnia symptoms in the short- and long-term (6 months post-treatment completion). In addition, publications will derive from analyses of secondary outcome variables of interest, such as the impact on relationship satisfaction and mental health.

## Discussion

This is the first randomised clinical trial to investigate the impact of including the bed partner in insomnia treatment. Given that insomnia is a prevalent and costly sleep disorder associated with poor mental and physical health outcomes and an increased risk of accidents, finding the most effective long-term treatment for insomnia is critical. This trial may optimise treatment outcomes for insomnia by improving adherence and reducing attrition. In addition, results will provide insight into the role partners play in clients’ insomnia presentation and help us better understand the impact of the partner on both treatment response and adherence in CBTI. Results could lead to the dissemination of a new intervention readily implementable in a wide range of clinics.

Overall, if our hypotheses are borne out and the partner-assisted version of CBTI improves adherence, and thus sleep outcomes, translation of PA-CBTI into clinics could have positive impacts beyond just sleep. Improvements in insomnia can have positive impacts on psychological health such as depression [56, 57] and anxiety [58]. Given the direct and indirect economic costs of insomnia, this study also holds potential to reduce overall healthcare costs and reduce one source of drag on economic output. It was estimated that in 2016–2017 the impact of inadequate sleep in Australia (population 24.8 million) was $45.21 billion [3].

### Limitations

A limitation of the current study is that neither clients nor clinicians are blinded to the treatment condition, a problem inherent to any intervention of psychotherapy. However, clients are blinded to the study hypotheses and staff who score polysomnography, actigraphy, and questionnaires are blind to treatment condition.

In addition, because we want to be reflective of a clinic-based population, and for the sake of feasibility and to reduce participant burden, the ‘baseline’ sleep diary and actigraphy data are collected between weeks 1–2 of treatment. Hence, this may not be considered as a true baseline. However, between weeks 1–2 the active intervention has not yet commenced as the first session consists of education only. Similarly, our ‘post-treatment’ sleep diary and actigraphy data are collected between weeks 6–7 of treatment. However, the final treatment session is a summary session only and no additional treatment strategies are introduced. All other post-treatment data (e.g. questionnaires) are collected after completion of treatment session 7.

We do not conduct a diagnostic assessment in the partners. Thus, it is possible some of them meet the criteria for a sleep disorder. While we considered conducting such screens, conceptually we do not believe the presence of a sleep disorder should have an impact on the partner’s ability to perform the role defined for them within the intervention. We do, however, have the STOP-Bang from screening, as well as Insomnia Severity Index, sleep diary, and actigraphy at each assessment time point. Thus, we can assess the risk of obstructive sleep apnoea and track symptoms of insomnia in the partners. If we have a couple where both partners report insomnia upon initial contact with the research team, we ask the couple to decide who will enrol as the “client” and who will enrol as the “partner.” While the partner is not required to adopt treatment recommendations themselves, they are welcome to change their behaviour in accordance with treatment if they so desire. This is true whether or not the partner reports sleep problems. Not having formal diagnostic information on the partners limits our ability to assess effectiveness of the interventions in couples where both individuals experience a diagnosed sleep disorder.

Finally, our lack of an individual SMT treatment condition could be considered a limitation of this trial. However, the decision not to include this group was made due to feasibility. An individual SMT group would have required a significantly larger sample allocated to a condition for which we do not expect to have favourable outcomes.

### Strengths

Strengths of the current project include the range of outcome variables, which permits high-quality analysis. Our focus on optimising external validity by maximising generalisability to clinical settings and implementing relatively few exclusion criteria is a significant strength, given clinic-based studies and clients with comorbidities show reduced adherence and completion relative to more tightly controlled RCTs. In addition, our control condition, sleep management therapy (PA-SMT), is a valuable control given that it is considered ‘treatment as usual’ in the community. Results could lead to the dissemination of a new CBTI intervention that helps more people adhere to and complete treatment, ultimately benefiting a greater number of people who seek treatment for insomnia. PA-SMT allows us to assess for the unique contribution of the partner independent of the active treatment. Another strength of this trial is our multimodal assessment of sleep; that is, both prospective (e.g. sleep diaries) and retrospective (e.g. ISI) data are collected. Furthermore, we control for clinician time and effort in that clinicians dedicate the same amount of time and effort to each of the three treatment conditions. This trial is also not limited to the investigation of how the interventions impact on sleep since data on broader outcomes will be analysed, including relationship functioning and mental health. Finally, this trial will allow a robust analysis of the impact of insomnia and its treatment on the bed partner, something not examined to date (Additional file 1).

## Trial status

Screening for participants began on 8 July 2016. The first enrolment was 6 September 2016. It is expected that enrolment will continue until July 2019.

## Additional file

Additional file 1: SPIRIT 2013 checklist: recommended items to address in a clinical trial protocol and page location within the Word version of the manuscript. (DOC 120 kb)

## Abbreviations

BAI: Beck Anxiety Inventory
CAF: Client adherence form
CBTI: Cognitive behavioural therapy for insomnia
CPQ: Communication Patterns Questionnaire
CSQ: Client Satisfaction Questionnaire
DAS: Dyadic Adjustment Scale
DUKE: Duke Structured Interview for Sleep Disorders
i-CBTI: Individual cognitive behavioural therapy for insomnia
ISI: Insomnia Severity Index
MANOVA: Multivariate analysis of variance
PA-CBTI: Partner-assisted cognitive behavioural therapy for insomnia
PA-SMT: Partner-assisted sleep management therapy
PCL-5: Posttraumatic Stress Disorder Checklist-5
PHQ-9: Patient Health Questionnaire-9
QLES-Q-SF: Quality of Life Enjoyment and Satisfaction Questionnaire Short Form
RCT: Randomised control trial
SC: Stimulus control
SRT: Sleep restriction therapy
